# Fabrication of conductive Ag/AgCl/Ag nanorods ink on Laser-induced graphene electrodes on flexible substrates for non-enzymatic glucose detection

**DOI:** 10.1038/s41598-023-48322-y

**Published:** 2023-11-28

**Authors:** Rana Bagheri, Saeid Alikhani, Ebrahim Miri-Moghaddam

**Affiliations:** 1 Department of Molecular Medicine, Faculty of Medicine, Cardiovascular Diseases Research Center, Birjand University of Medical Sciences, Birjand, 9717853577 Iran; 2 Nanofanavaran partopooyesh Company, Science and Technology Park of South Khorasan, Birjand, 9718643683 Iran

**Keywords:** Biomarkers, Nanoscience and technology

## Abstract

An unusual strategy was designed to fabricate conductive patterns for flexible surfaces, which were utilized for non-enzymatic amperometric glucose sensors. The Ag/AgCl/Ag quasi-reference ink formulation utilized two reducing agents, NaBH$_{4}$ and ethylene glycol. The parameters of the ink, such as sintering time and temperature, NaBH$_{4}$ concentration, and layer number of coatings on flexible laser-induced graphene (LIG) electrodes were investigated. The conductive Ag/AgCl/Ag ink was characterized using electrochemical and surface analysis techniques. The electrocatalytic activity of Ag/AgCl/Ag NRs can be attributed to their high surface area, which offer numerous active sites for catalytic reactions. The selectivity and sensitivity of the electrodes for glucose detection were investigated. The XRD analysis showed (200) oriented AgCl on covered Ag NRs, and with the addition of NaBH$_{4}$, the intensity of the peaks of the Ag NRs increased. The wide linear range of non-enzymatic sensors was attained from 0.003 to 0.18 mM and 0.37 to 5.0 mM, with a low limit of detection of 10 $\upmu$M and 20 $\upmu$M, respectively.The linear range of enzymatic sensor in real sample was determined from 0.040 to 0.097 mM with a detection limit of 50 $\upmu$M. Furthermore, results of the interference studies demonstrated excellent selectivity of the Ag/AgCl/Ag NRs/LIG electrode. The Ag/AgCl/Ag NRs on the flexible LIG electrode exhibited excellent sensitivity,long-time stablity,and reproducibility. The efficient electroactivity were deemed suitable for various electrochemical applications and biosensors.

## Introduction

The fabrication of a typical flexible electronic device requires the patterned deposition of component materials on a flexible substrate. Conductivity inks are generally prepared using particles such as silver, copper, gold, graphene, and carbon nanotubes^1–4^. The microfabrication of conductive patterns on flexible surfaces has recently become increasingly noticeable^5^. For the fabrication of electronic devices, materials are often deposited using solvents such as water and organic solvents such as ethylene glycol, toluene, and cyclohexane^6^. Silver is the best material for use as a conductive ink and adhesive compared to other electrically conductive fillers owing to its high thermal and electrical conductivities, chemical stability, relatively low cost, and low melting point for thin film formation on flexible surfaces. Different methods can be used to synthesize and stabilize silver nanoparticles^7–9^. One of the most popular methods is chemical reduction, using a variety of organic and inorganic reducers^10–12^. Depending on the method used, Ag can be prepared with different morphologies, sizes, and shapes, which highly depend on the nucleation and development of Ag salt reduction^13^. In addition, various parameters can affect the nucleation and growth of silver nanoparticles, including temperature^14^, pH of precursors^15^, protective agents^16^, and stabilizing agents^17^. Silver ink with NaCl and KCl agents was fabricated as quasi-reference electrodes. Recently, researchers reported various methods for the synthesis of Ag/AgCl nanocomposites^18,19^, AgCl-Ag@AgCl nanowires^20^, and Ag/AgCl nanoparticles^21^. In 2014, laser-induced graphene (LIG) from polyimide (PI) films was studied using a one-step process for energy storage and electrode surface application^22^. Moreover, the researchers achieved a well-established technique for flexible electrodes based on porous few-layer graphene, which can be made in one stage by using a CO$_{2}$ laser scriber on PI polymers^23,24^. This electrode has low resistance and high conductivity and can be used in the construction of batteries as a substrate for electrodes. Furthermore, these electrodes do not need a mask, and the design is created with high accuracy^25^. The conductive LIG electrode can perform in terms of patterns, such as a single-strip three-electrode system and microfluidic electrode to the quasi-reference electrode prepared using conductivity Ag/AgCl ink^26^. The formation of Ag/AgCl NRs is a complex process involving the interplay of various chemical and physical factors. However, by carefully controlling these factors, it is possible to synthesize Ag/AgCl NRs with a high degree of control over their size and shape, which is important for potential applications in various fields, including catalysis, sensing, and biomedical imaging^27,28^. Recently, the detection of targets such as chloride, humidity, and H$_{2}$O$_{2}$ on Ag/AgCl substrates has also been investigated^29–31^. However, non-enzymatic glucose sensors on Ag/AgCl/Ag NRs ink on three-dimensions (3-D) graphene structures have not yet been investigated. Many different types of nanomaterials can be used to enhance the surface of the electrodes. In the case of electrochemical glucose detection sensors, metallic nanoparticles of varying sizes and morphologies have been found to improve the linear range and limit of detection (LOD) of sensors^32–36^. Recently, metallic nanoparticles such as copper^37^, platinum^38^, prussian blue (PB)^39^, silver^40–42^, and gold^43–45^ have been used for this purpose. Flexible glucose sensors have shown great potential in revolutionizing diabetes management by providing real-time insights into blood glucose dynamics and trends^46^. The continuous glucose monitoring (CGM) devices have been proven to improve the safety and effectiveness of diabetes therapy by reducing hypoglycemia incidence and duration while decreasing glycemic variability^47^. Recently, researchers examined the use of mobile and wearable technology for monitoring diabetes-related parameters^48^. CGM are considered flagship technologies in biosensor research. Further research is needed to improve the accuracy and stability of non-invasive glucose sensing and to develop validated clinical trials for wearable glucose monitoring systems^49^. Recently, researchers have been paying special attention to the process of glucose measurement techniques with non-enzymatic-based invasive, minimally invasive, and non-invasive approaches^49^. The major challenge in glucose sensing is the interference caused by temperature variations and binding-induced changes^50^. This issue becomes particularly critical in wearable glucose monitoring devices, where the sensor is subjected to various mechanical deformations due to body movements. To address these challenges, researchers are actively working on developing sensors that are independent of environmental conditions (e.g., temperature or relative humidity) or upon mechanical deformations (e.g., stretching, bending, or twisting). These sensors utilize advanced materials and designs that can minimize the impact of different conditions on glucose measurements. There is always a need to make attractive and alternate strategies to monitor glucose with many advantages like simplicity, low cost, stability, and high sensitivity^51^.For example, nanomaterial-based sensors have shown promise in providing glucose measurements by utilizing the unique properties of nanomaterials^52^.

LIG as flexible electrodes are known for their high stability and excellent electrical conductivity, which make them ideal substrates for electrodes. The use of Ag/AgCl/Ag NRs on the LIG electrodes can further enhance the selectivity and sensitivity of glucose detection.

When a sample containing glucose is introduced to the sensor, the mediator then transfers the electrons to the Ag/AgCl/Ag electrode, generating a current that is proportional to the glucose concentration in the 0.1 M NaOH solution using the amperometric technique. The advantages of using an Ag/AgCl/Ag substrate for glucose electrochemical sensors include electrocatalysis, high sensitivity, selectivity, reproducibility, and low cost. Ag/AgCl electrodes are commonly used in electrochemical measurements because of their high conductivity and low resistivity. This study aimed to develop different conductive formulations of Ag/AgCl/Ag ink, investigate their properties, and evaluate the performance of the fabrication process. We prepared a new type of Ag/AgCl/Ag ink using a reduction method that utilized NaBH$_{4}$ and ethylene glycol (EG). NaBH$_{4}$ and EG can be used as reducing agents to synthesize the Ag/AgCl/Ag NRs. NaBH$_{4}$ can reduce silver ions to form silver nanostructures, which can then react with chloride ions to form Ag/AgCl NRs. EG can act as a solvent and a reducing agent to form Ag/AgCl NRs. This study determined the performance of different conductive reference inks by studying the electrochemical properties of the LIG electrodes, XRD, DLS, TGA, four-point probe resistivity surface, UV-Vis absorption spectrum, and SEM. Various sintering techniques are typically used, such as thermal, chemical, electric, and laser sintering after the printing process. In this study, the thermal sintering process at different times and temperatures was investigated, and a three-electrode system was used for glucose detection. The prepared flexible electrodes were developed using a low-cost, simple fabrication process. The excellent sensitivity and selectivity of glucose electrochemical sensors on Ag/AgCl/Ag NRs/LIG substrates under optimized conditions were investigated. The results revealed that the proposed electrode can be widely used in clinical and research settings for electrochemical applications and for the detection of glucose levels in the blood, urine, and other biological samples.

## Results and discussion

### Electrochemical characterization of the ink on LIG electrodes

**Figure 1:**
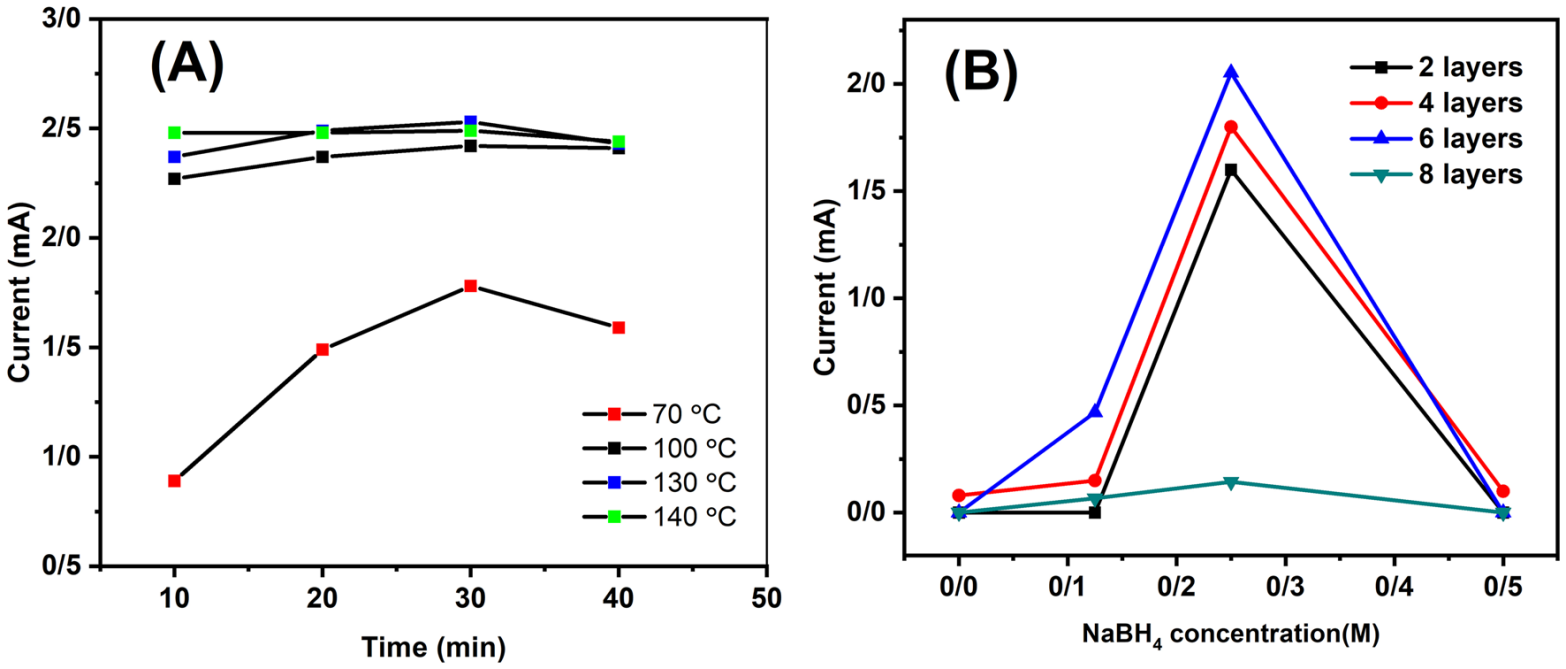
(**A**) Effects of sintering temperature and time, (**B**) NaBH$_{4}$ concentration, and various layers of Ag/AgCl/Ag ink.

For the characterization of the prepared electrodes, cyclic voltammetry (CV) measurements were carried out to verify the performance of each electrode pair using a 5.0 mM K$_{3}$[Fe(CN)$_{6}$], 5.0 mM K$_{4}$[Fe(CN)$_{6}$], 0.1 M KCl solution and 5.0 mM K$_{3}$[Fe(CN)$_{6}$], 0.1 M KNO$_{3}$ in phosphate-buffered saline (PBS; pH 7.4) solution as an electrolyte. The electrochemical measurements were repeated three times. In Online Resource Fig. S2A, the reduction of AgCl to Ag is a common electrochemical reaction that can occur on Ag/AgCl/Ag electrodes in cyclic voltammetry. This reaction is typically observed as a reduction peak in cyclic voltammograms. The reduction of AgCl to Ag occurs when the potential of the electrode is more negative than that of AgCl. At this point, electrons are transferred from the electrode to AgCl, reducing it to Ag. The reduction potential of AgCl depends on several factors, including the concentration of Cl$^{-}$ ions in the electrolyte solution. However, it is important to note that the reduction of AgCl can interfere with other electrochemical reactions of interest. The oxidation peak near 0.5 V corresponds to the formation of AgCl on the electrode surface, due to redox reactions of silver ions with chloride ions of potassium chloride in the electrolyte and decreased electron transfer in the cyclic voltammetry response. To minimize the interference of AgCl reduction in cyclic voltammetry experiments, the concentration of Cl$^{-}$ ions in the electrolyte solution was minimized. In Online Resource Fig. S2B, was determined, the exact peak was not shown, and the values of $\Delta$E were emitted about 0.1 V which is consistent with another reports^53,54^. The ink was the painted onto the electrode surface. The conductivity of the Ag/AgCl/Ag surface with a light-emitting diode (LED) was shown in Online Resource Fig. S2C. Initially, an amperometric technique was used to study and optimize the electrode performance (Online Resource Fig. S3). The amperometric responses were investigated at various potential ranges from +300 to +600 mV vs. Ag/AgCl/Ag. The response current increased as the potential increased from +300 mV to +400 mV, and then decreased at potentials higher than +400 mV. The maximum current was obtained at +400 mV, and was used for subsequent experiments. Figure 1A shows the effect of sintering temperature and time on the current response of the Ag/AgCl/Ag ink on the electrode surface. The sintering process reduces the path of contact between the conductive particles and the volume of the matrix through baking or evaporation, resulting in increased conductivity between the Ag/AgCl/Ag particles. The results indicate that increasing the sintering temperature from 70 to 140 $^\circ$C contributes to an increase in current and the response at the sintering temperature of 140 $^\circ$C was approximately three times higher than that at 70 $^\circ$C. There was no significant difference in the response between 100 and 140 $^\circ$C, and the current of the Ag/AgCl/Ag layers increased slightly. The electrical current of Ag/AgCl/Ag layers was increased from 10 to 30 min, and then, with a further increase in the sintering time, the current response decreased. Therefore, the most suitable sintering parameters were achieved at 130 $^\circ$C for 30 min to obtain a high current response in the Ag/AgCl/Ag layer. Ag/AgCl/Ag NRs were prepared using a reduction method with NaBH$_{4}$ as the reducing agent. The NaBH$_{4}$ concentration is an important parameter for the synthesis of metal nanoparticles. The NaBH$_{4}$ concentration varied in the range of 0–0.5 M (Fig. 1B). As the concentration increased up to 0.25 M, the response also increased but began to decrease. Therefore, 0.25 M NaBH$_{4}$ was used in the reduction process. The effect of the number of layers between two and eight on the current response was analyzed (Online Resource Fig. S4). The highest response was obtained at six layers, which can be attributed to the increased contact area of Ag/AgCl/Ag and improved electrode performance. However, when the number of layers is greater than six, the thickness of the ink on the LIG electrode causes a decrease in the electrode response.

**Figure 2:**
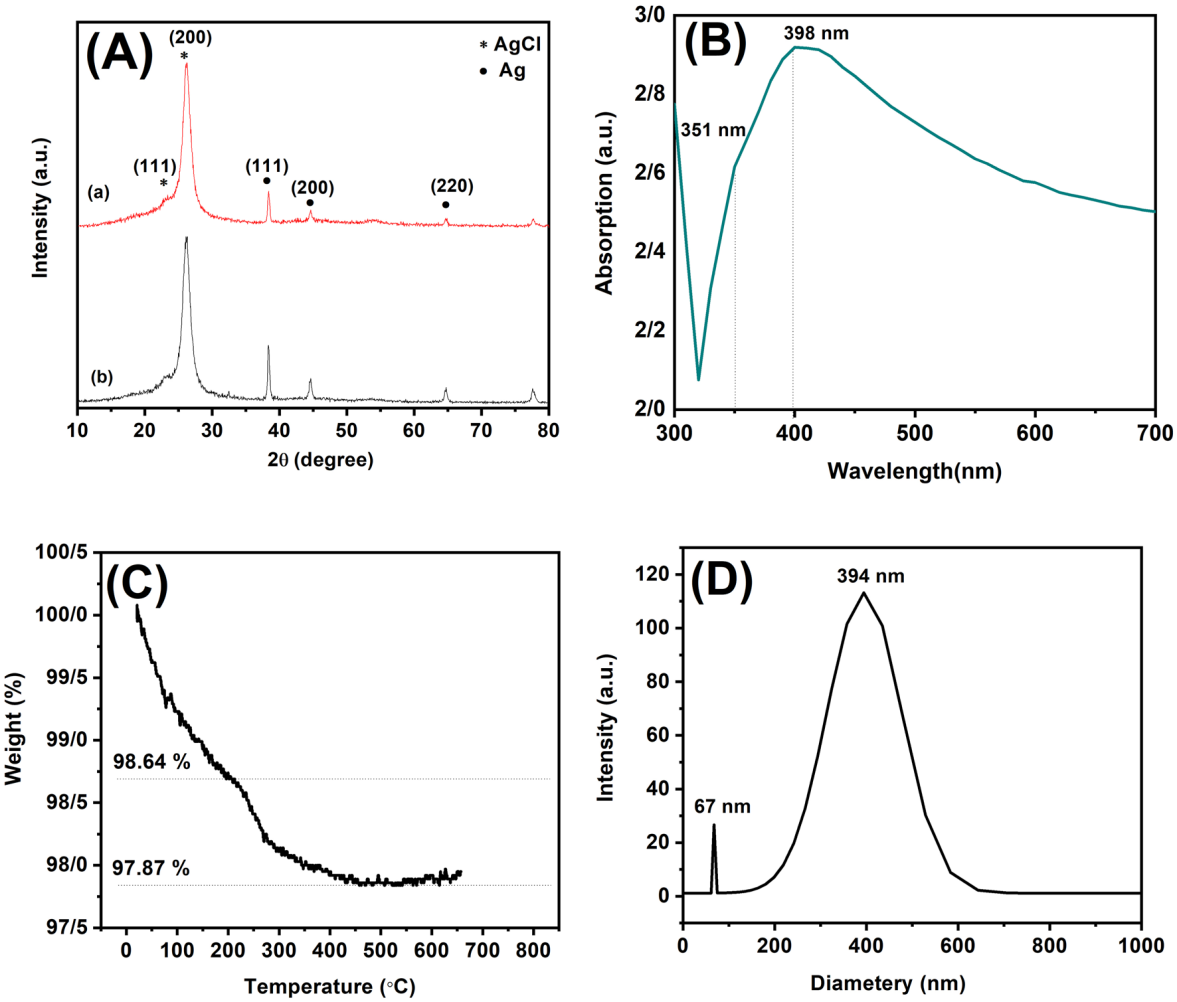
(**A**) XRD patterns of Ag/AgCl/Ag Nanorods synthesized at 0 M (**a**) and 5.0 M (**b**) NaBH$_{4}$, (**B**) UV–Vis absorption spectrum, (**C**) Thermogravimetric analysis, (**D**) The DLS analysis of Ag/AgCl/Ag ink.

**Figure 3:**
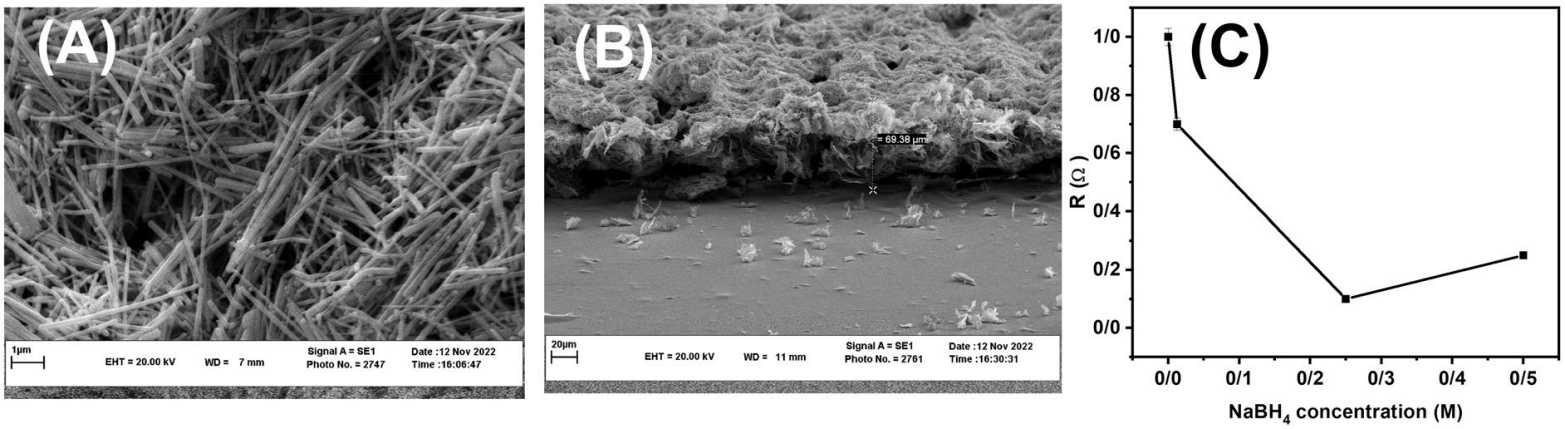
(**A**) SEM image, (**B**) cross-sectional, and (**C**) curve of resistivity versus various NaBH$_{4}$ concentrations of Ag/AgCl/Ag on the LIG substrate.

NaBH$_{4}$ was used to synthesize Ag/AgCl/Ag NRs. However, the presence of NaBH$_{4}$ can also affect the XRD analysis of the Ag/AgCl/Ag NRs. To understand the role of NaBH$_{4}$ in the formation of the Ag/AgCl/Ag layer, the reactions were performed in the presence and absence of NaBH$_{4}$. XRD analysis was perform to obtain information regarding the crystal structure and phase composition of the material. The diffraction peaks for silver were broad and diffuse, indicating that silver was polycrystalline (Fig. 2A(a)). On the other hand, the diffraction peaks for AgCl, were sharper and more intense, indicating that silver chloride was in a more crystalline form. The XRD pattern was used to determine the crystal structure of the AgCl phase, which was found to be cubic. The diffraction peaks were indexed at 2$\theta$= 23.10, 26.02, and 53.69 assigned to the (111), (200), and (311) facets respectively, revealing the preferred growth of AgCl along (200) with the highest intensity. However, some differences were observed at 2$\theta$= 38.16, 44.22, and 64.57 according to the (111), (200), and (220) facets, indicating silver has the fine crystallinity. The results showed that the intensity of the (111) characteristic peaks of face-centered cubic Ag increased (1.4 times) with the addition of NaBH$_{4}$ (Fig. 2A(b)). This indicates that in the presence of NaBH$_{4}$, chloride ions could increase the reaction rate of growth of Ag NRs and increase the formation of Ag coating on the Ag/AgCl NRs surface. In the presence of KCl, AgCl covers to faces of an Ag NR core^55^. The Ag NRs growth formed along (200). The AgCl diffraction pattern of Ag NRs is observed, resulting in high crystallinity of AgCl. The peaks of the Ag cubic and AgCl cubic are present at the same time in Ag/AgCl NRs, indicating that Cl is replaced on the surface of Ag NRs. The results were consistent with other reported study^20^. Ag ions were reduced to Ag NRs by EG and PVP, and nucleation led to the growth of rod-shape particles with a PVP protective agent. Additionally, a (200) AgCl layer fabricated by the precipitation method adhered with a suitable PVP concentration to the surface of Ag NRs to form an AgCl shell^16^. To achieve preferential orientation of the (200) facets, a protective agent such as PVP was added to the solution. PVP preferentially binds to the (100) facets of the silver NRs, which slows the growth of the (100) facets and promotes the growth of the (200) facets. The presence of NaBH$_{4}$ can affect the crystal structure and lattice parameters of Ag/AgCl/Ag NRs, which can, in turn, affect their XRD patterns. Specifically, the reduction of silver ions by NaBH$_{4}$ can lead to the formation of a metallic silver nanostructure, which can coexist with the Ag/AgCl NRs in the sample, and the Ag peak intensity increases. Subsequently, the formed AgCl shell was decorated with Ag nanoclusters, and the Ag NRs were increased in the suspension ink. The average crystallite size of absent and present has obtained 10.40 nm and 0.45 nm, respectively, which indicated the effect of NaBH$_{4}$ on lower crystallite size. The UV-Vis absorption spectrum of the ethanol and acetone solutions of the Ag/AgCl/Ag ink exhibited a shoulder peak at a wavelength of 351 nm, which emphasized the Ag/AgCl/Ag NR structures (Fig. 2B). Another peak was observed at 398 nm, indicating the oscillation of conduction band electrons as surface plasmon resonance (SPR)^56^. The particle size, aspect ratio, and diameter of the nanorods effect the SPR wavelength. The results indicate that the prepared Ag/AgCl/Ag ink contained two particle distributions. The TGA profiles of the Ag NRs powders with different particle sizes washed three times were obtained. The Ag NRs were centrifuged at 8000 rpm for 30 min, and the resulting NRs were used to form Ag/AgCl/Ag NRs paste, which was then heated in an oven at 130$^\circ$C for 30 min and washed with water at 70$^\circ$C for 10 min. This process was repeated thrice to remove the PVP solvent. Figure 2C shows TGA curves of the Ag/AgCl/Ag nano powder after washing three times. Several endothermic and exothermic reactions were detected in N$_{2}$, and the Ag/AgCl/Ag NRs showed continuous weight loss up to 200$^\circ$C. The continuous weight loss from the initial temperature to 200$^\circ$C can be attributed to the evaporation of low-and high-boiling-point solvents and dispersants, respectively. Another significant weight loss was observed in the temperature range 200–425 $^\circ$C. Under a N$_{2}$ atmosphere, the mass loss upon heating to 425 $^\circ$C was 2.05%. Thus, the PVP-to-Ag weight ratio of the Ag/Ag NRs was calculated. The results showed that the weight loss of PVP at 200–425 $^\circ$C was 0.77%. The PVP-to-Ag weight ratio was 0.007, indicating that the Ag/AgCl/Ag ink was successful fabricated. This PVP content in the ink was much lower than that reported in similar^57^. In during the temperature range where PVP decomposition occurred, Ag/AgCl/Ag NRs could serve as a catalyst for the decomposition and combustion of organic molecules, and these organic molecules were attached to the particles, which were linked together as a net^58,59^. Figure 2D shows the formation of the Ag/AgCl/Ag ink and the mean hydrodynamic diameters of the particles obtained by DLS analysis. The mean particle size of 394 nm and another peak at 67 nm indicate that the nanostructure directly interfered with the quality of the formed NRs. It is desirable to control the nanorod aspect ratio using an AgCl/Ag ink with a PVP monomer^53^. Figure 3A illustrates the SEM images of the synthesized Ag/AgCl/Ag NRs, indicating a high degree of uniformity in diameter along each rod, with a mean diameter of approximately 25 nm and a length of 1.3–5 $\upmu$m, which is consistent with the UV–Vis absorption spectrum results. Figure 3B shows cross-sectional SEM images of Ag/AgCl/Ag NRs grown on a LIG substrate. High-density uniformly distributed NRs were grown on the electrode substrate. The thickness of the coating is approximately 48.59 $\upmu$m. The relationship between surface resistivity and various NaBH$_{4}$ concentrations ranging from 0 to 0.5 M was systematically investigated using four-probe surface resistivity measurements (Fig. 3C). The results showed that the surface resistivity decreased with increasing NaBH$_{4}$ concentration until it reached a minimum value of 0.002 $\Omega$ cm$^{-2}$, after which it increased. The addition of NaBH$_{4}$ until 0.25 M to the ink also increased the reduction of Ag ions and decreased the resistivity, resulting in an increased current response, which is consistent with the amperometric results. In addition, NaBH$_{4}$ can affect the size, shape, and stability of the Ag/AgCl/Ag NRs. The addition of NaBH$_{4}$ concentration to 0.25 M can lead to a higher density of Ag/AgCl/Ag NRs on the substrate, resulting in lower surface resistivity. However, if the concentration of NaBH$_{4}$ is higher than 0.25 M, it can lead to the formation of larger and more irregularly shaped NRs, which can reduce the surface area and increase the surface roughness of the substrate, leading to a higher surface resistivity. Therefore, the optimal concentration of NaBH$_{4}$ (0.25M) for achieving the lowest surface resistivity of the Ag/AgCl/Ag nanorod substrate was determined.

### Mechanism of glucose sensor-based Ag/AgCl/Ag on LIG electrode

silver NRs have been extensively studied for their potential applications in the electrochemical detection of non-enzymatic glucose. This is due to their excellent electrocatalytic activity towards glucose oxidation, which makes them promising candidates for glucose sensing. The mechanical detection of glucose using Ag/AgCl/Ag NRs involves the oxidation of glucose to gluconic acid and the reduction of AgCl to Ag. The oxidation of glucose occurred at the surface of the Ag/AgCl/Ag NRs, which acted as catalysts for the reaction. Simultaneously, the reduction of AgCl to Ag results in the formation of metallic Ag nanostructures on the surface of the NRs. The presence of metallic silver nanostructures on the surface of the Ag/AgCl/Ag NRs enhanced their electrocatalytic activity towards glucose oxidation. This is because the metallic Ag nanostructure provides additional active sites for glucose oxidation, which increases the efficiency of the reaction. The electrochemical detection of glucose involves the use of a three-electrode system with Ag/AgCl/Ag NRs serving as the working electrode. Glucose concentration was determined by measuring the current generated by the oxidation of glucose on the surface of the NRs.

#### The electrochemical non-enzymatic glucose sensor

To demonstrate the non-enzymatic glucose detection performance of the Ag/AgCl/Ag NRs electrode, various glucose concentrations were prepared in 0.1 M NaOH (Online Resource Fig. S5). The potential range was chosen from $-100.0$ to 100.0 mV, at a scan rate of 50 mV s$^{-1}$. In the absent glucose, The two anodic peaks observed in the cyclic voltammograms can be attributed to the electroformation of Ag$_{2}$O multilayer, and the oxidation of Ag$_{2}$O to AgO, respectively. Similarly, the two cathodic peaks can be attributed to the reduction of AgO to Ag$_{2}$O and the further reduction of Ag$_{2}$O to Ag, respectively^60^. Regarding the interaction with chloride, it is indeed likely to reduce the dissolution of the AgNPs and increase their persistence^61^. However, it is important to note that the oxidative dissolution of Ag/AgCl/Ag NRs can be attenuated by biological molecules such as glucose. The oxidation current in the cyclic voltammetry experiments can increase in the presence of glucose on the Ag/AgCl/Ag substrate. This is because glucose can act as a reducing agent, donating electrons to the Ag/AgCl/Ag electrode and increasing the rate of oxidation. The increase in oxidation current can be used to quantify the concentration of glucose in the electrolyte solution.

#### Optimization of analytical parameters

Initially, the performance of sensor was investigated at various potential ranges from +200 to +500 mV vs. Ag/AgCl. The response current increased as the potential increased from +200 to +400 mV ( Online Resource Fig. S6A). It is worth noting that the applied potential of the electrode for glucose detection was lower compared to other reports, glucose oxidation occurred at a lower potential^62,63^. The pH of the solution can have a significant effect on the electrochemical detection of non-enzymatic glucose by Ag/AgCl/Ag, as it can significantly affect the electrocatalytic activity towards glucose oxidation. The CV response showed that two anodic peaks attributed to the formation of Ag$_{2}$O and AgO in the 0.1M NaOH solution, respectively. Based on the observed glucose interaction in CV, the performance of electrode was evaluated in various pH conditions ranging from 8 and 14 using amperometric measurements at 400 mV, corresponding to the current generated in AgO. The results are presented in Online Resource Fig. S6B. At lower pH values, Ag/AgCl/Ag NRs may undergo dissolution, which can lead to a decrease in their electrocatalytic activity toward glucose oxidation. This is because the acidic environment can cause Ag/AgCl/Ag NRs to dissolve, resulting in the loss of active sites for glucose oxidation. At higher pH values, the Ag/AgCl/Ag NRs exhibited improved electrocatalytic activity for glucose oxidation (pH >12). The optimal pH can vary depending on the specific synthesis method and reaction conditions used and is typically determined experimentally. The optimal pH for glucose detection using Ag/AgCl/Ag NRs was typically in the range of 14.The high sensitivity observed in alkaline solution may be attributed to the other nanostructure substrates^33^. The flexibility of the Ag/AgCl/Ag/LIG electrode on a flexible PET substrate was evaluated through an outer bending test ( Online Resource Fig. S7A). The change in the current resulting from bending was expressed as (I-I $_{0}$ )/I$_{0}$ , where I$_{0}$ is the initial measured current for a flat electrode and I is the current measured under substrate bending. The electrode exhibited a changed in current of 13% for a bending radius of 10 mm, indicting its intrinsic flexibility for glucose detection. Furthermore, the effect of temperature on the proposed sensor was studied, ranging from 27 to 52 $^\circ$C (Online Resource Fig. S7B). The results showed a temperature changed of approximately 20% at 52 $^\circ$C . It is worth noting that a non-glucose sensor with Ag/AgCl/Ag NRs/LIG electrode was found to be insensitive to both bending and temperature variations. To investigate the simultaneous action of glucose and strain, when strain is applied to the sensor, the porous structure of electrode compresses, leading to a noticeable increase in current.The results are presented in Online Resource Fig. S8. Once the strain is removed, the film returns to its original geometry and position. The Ag/AgCl/Ag ink applied to the LIG electrode fills most of the porous structure of 3-D graphene, allowing the electrode can be used to fabricate a bending-insensitive electrode^64^. In the present of ink on graphene to be used for fabricating a bending insensitive electrode. The presence of the ink on the graphene helps maintain its initial conductivity. However, due to the slight change in structure and the penetration of the ink into the porous structure, there is a slight increase in the current rate at each stage.

#### Analytical performance of proposed glucose sensor

**Figure 4:**
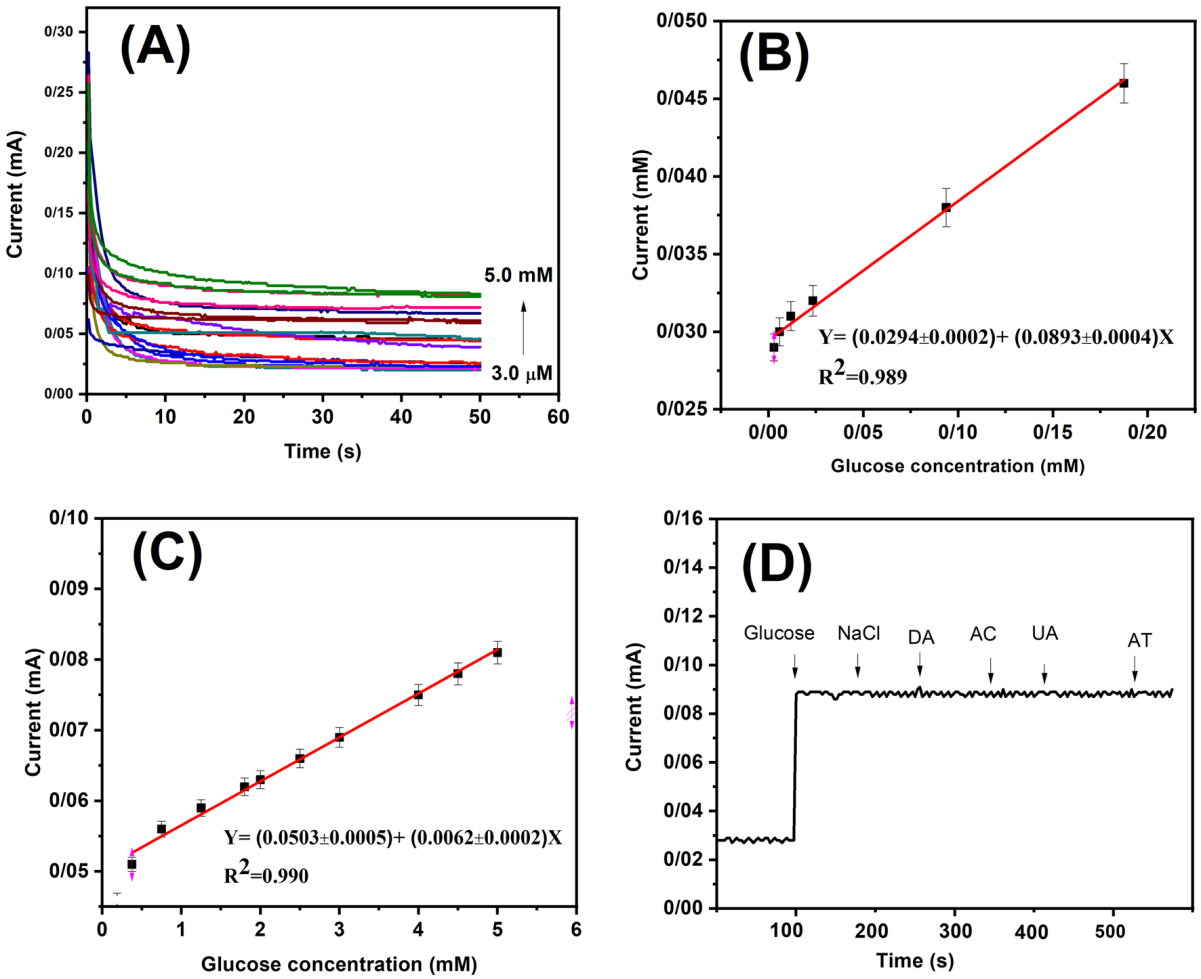
(**A**) Amperometric responses of Ag/AgCl/Ag/LIG electrode for various glucose concentrations (0.003–5.0 mM) in 0.1 M NaOH at +400 mV. The calibration curve of current response versus glucose concentrations with linear ranges: (**B**) 0.003–0.18 mM, (**C**) 0.37–5.0 mM, (**D**) Current response of Ag /AgCl NRs/ LIG electrode in 0.1 M NaOH upon successive additions of 5.0 mM glucose, 0.1 mM NaCl, DA, AC, UA, and AT at +400 mV applied potential.

The amperometric detection of different glucose concentrations using a non-enzymatic Ag/AgCl/Ag electrode on a LIG substrate was employed (Fig. 4A). A steady current from 20 s was used as the current response, and the current increased with increasing glucose concentration, as shown in Online Resource Fig. S5. The calibration curves (Fig. 4B,C) show two linear ranges: 0.003–0.18 mM and 0.37–5.0 mM, with a limit of detection of 10 $\upmu$M and 20 $\upmu$M, respectively. The corresponding linear equations were I (mA) = (0.02940 ± 0.002) + (0.08930 ± 0.004) C (R$^{2}$ = 0.989) and I (mA) = (0.05030 ± 0.005) + (0.00620 ± 0.002) C (R$^{2}$ = 0.990). It is desirable for the sensitivity at high glucose concentrations to be lower than that at lower concentrations because at high concentrations, all of the available binding sites on the sensor become occupied, leading to a plateau in the signal. This can result in a decrease in sensitivity, as the system is no longer able to distinguish between different concentrations of the analyte. The performance of non-enzymatic glucose sensor in real sample were evaluated by determining the glucose concentration in serum sample (The results are presented in Online Resource Fig. S9). The calibration curve shows linear range between 0.040 and 0.097 mM with a limit of detection of 50 $\upmu$M. The corresponding linear equations were I (mA) = - (0.064 ± 0.006) + (2.500 ± 0.094) C , (R$^{2}$ = 0.99142).

#### Selectivity, long-term stability and reproducibility of the glucose sensor

The selectivity of the proposed sensor was investigated in the presence of interfering agents in 0.1 M NaOH. Figure 4D shows the amperometric responses of the interfering agents, including NaCl, Dopamine (DA), Ascorbic acid (AC), Uric Acid (UA), and Acetaminophen (AT). In the presence of glucose, the current increased; however, the interfering agents had no effect the current, indicating the selectivity of the proposed sensor for electrochemical glucose detection. The study of interfering agents was conducted by adding 100 $\upmu$M at an applied voltage of +400 mV. Repeatability characterizes the consistency and precision of measurements obtained from the same sensor under similar operating conditions.The Ag/AgCl/Ag NRs/LIG multiple electrodes were tested, that showed negligible current loss during testing. This suggests that the manufacturing process for the electrodes is reliable and can be repeated with consistent results. The results shown in Fig. S10 further support the repeatability of the measurements obtained from these electrodes.The non-enzymatic glucose sensor also was determined stable for one week at room temperature (The results are presented in Online Resource Fig. S11). A summary of the performance of various non-enzyme glucose sensors is shown in Table 1, which indicates that the proposed detection method has good and comparable results to those of other methods.

**Table 1:** Performance of various non-enzyme glucose sensors using electrochemical techniques.

Sensing materials	Analytical method	Sensitivity ($\upmu$A mM$^{-1}$ cm$^{-2}$)	Linear rang (mM)	Limit of detection (mM)	References
ZnO/C/GCE	Cyclic voltammetry	2.97	0.1–10	1	^66^
SiO$_{2}$/C/CuO	Amperometry	472	0.02–20	0.06	^67^
PtNi alloy/G/GCE	Amperometry	24.03	0.5–15	0.016	^68^
Pt black-modified microneedles	Choroamperometry	145.33	1–30	0.48	^69^
Au/GO/GCE	Amperometry	4.56	0.1–2	0.025	^70^
Cu/RGO/SPCE	Choroamperometry	172	0.1–12.5	0.065	^71^
Ag/AgCl/Ag NRs/LIG	Amperometry	1.15	0.003–0.18	0.01	This work

## Conclusion

We developed a glucose microsensor on a LIG-based electrode using Ag/AgCl/Ag NRs. In this study, we prepared NRs under optimal conditions using a two-step chemical reduction method with PVP as a protective agent. To control the shape and size of the NRs, we carried out the process under optimal conditions, such as the reducing agent concentration, sintering temperature, and time. A low surface resistance of 0.002 $\Omega$ cm$^{-2}$ was obtained, indicating a high surface conductivity. Notably, the NRs interacted well with the porous structure of the LIG. The presence of AgCl (200) facets in the NRs enhanced their electrocatalytic activity; therefore, the proposed sensor was utilized as an electroactive material on a flexible LIG layer using an amperometric technique. The performance of the proposed sensor was investigated under optimal conditions, such as pH and applied potential. The low detection limit of 10 $\upmu$ M and 20 $\upmu$ M were obtained in wide linear range of 0.003–0.18 mM and 0.37–5.0 mM in 0.1 M NaOH, respectively, and limit of detection of 50 $\upmu$M was determined in real sample. The Ag/AgCl/Ag NRs on the flexible LIG electrode exhibited excellent sensitivity, long-time stablity, and reproducibility. Furthermore, this porous microelectrode has great potential for immobilizing metal NRs for electrochemical and biosensors applications.

## Methods

### Preparation of conductive Ag/AgCl/Ag nanorod ink

Ag/AgCl/Ag nanostructures were prepared by the chemical reduction of AgNO$_{3}$ by NaBH$_{4}$ in the PVP solution. Initially, 0.5 gr PVP and 0.2 gr KCl in EG (20 ml of EG were mixed at 130 $^\circ$C for 1 h) because PVP and EG did not dissolve at room temperature. KCl was used as the directing agent to control the formation of multiple Ag nucleations^53^. The PVP values must always be in excess because they lead to the growth of metal nanowires^16^. EG was used in the Ag/AgCl/Ag ink formulation to prevent the inks from drying and to increase the viscosity^65^. Then a 0.5 M of fresh AgNO$_{3}$ solution was added dropwise to the reaction mixture containing PVP and KCl for 4 h at 130 $^\circ$C and the solution color changed from yellow to white, indicating the formation of AgCl particles, the change is shown in Online Resource Fig. S1. Then 5.0 mL of NaBH$_{4}$ (0.25 M) solution was added to the prepared solution, and the resulting color immediately changed to yellow/brown. Finally, the Ag/AgCl/Ag conductive nanostructures were separated by centrifugation (8000 rpm for 30 min at 25 $^\circ$C) and washed twice with acetone and three time with ethanol.

### Preparation of Ag/AgCl/Ag ink on LIG surface electrode

**Figure 5:**
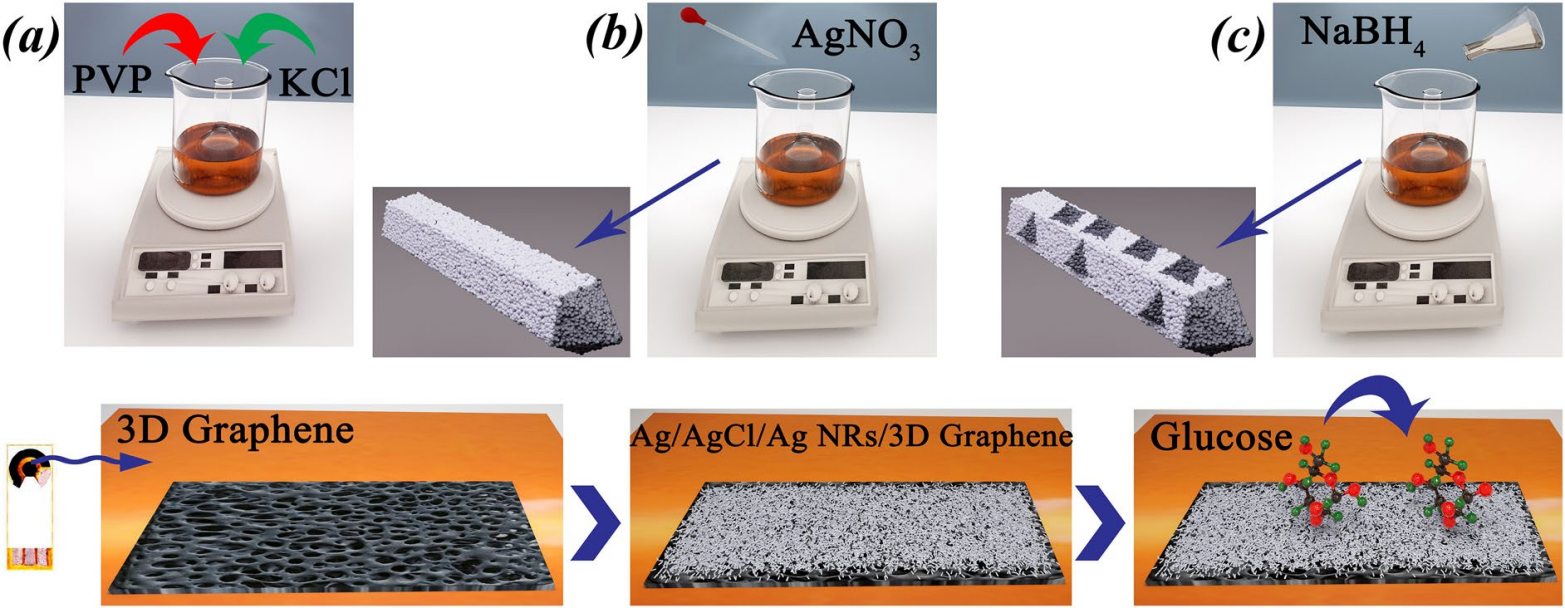
Schematic illustration of (up) preparation of Ag/AgCl/Ag ink on LIG surface electrode and (down) the fabrication of a non-enzymatic glucose sensor based on the Ag/AgCl/Ag NRs modified LIG.

A PI film, Kepton, with a thickness of 125 $\upmu$m was used as a flexible electrode substrate. First, the PI films were cleaned with ethanol and washed with acetone and DI water to remove surface contaminants. After the film was prepared, it was immersed in an aqueous KOH (9.0 M) solution to activate the carbon surface. After immersion, the sheets were dried in a vacuum oven at 60 $^\circ$C for 40 min. the LIG electrode patterns were designed using AutoCAD software, and the geometrical area (96 mm$^{2}$) of the film was cut. The PI sheets were then fixed on a PET slide. During the laser beam irradiation process in ambient air, the laser power, scanning speed, line spacing, and defocus were set at 25.5 W, 365 mm s$^{-1}$, 0.03 mm, and $-3.0$ mm respectively. Ag/AgCl/Ag ink was painted on 3-D graphene, and the prepared electrode was immersed in warm water (60–70 $^\circ$C) for 10 min or subjected to thermal sintering. The optimized sintering time and temperature were set to 130 $^\circ$C and 30 min, respectively. This step helped remove the PVP solution from the Ag/AgCl/Ag ink, thereby improving the conductivity. The preparation process of ink on LIG electrode for non-enzymatic glucose detection is shown in Fig. 5.

### Supplementary Information

Supplementary Information.

## Data Availability

The datasets analyzed in the current study are available from the corresponding authors upon request.
