# Supplementary material for: Fabrication of conductive Ag/AgCl/Ag nanorods ink on Laser-induced graphene electrodes on flexible substrates for non-enzymatic glucose detection
Source: Sci Rep. 2023 Nov 28;13:20898. doi: 10.1038/s41598-023-48322-y (PMC10684547; doi:10.1038/s41598-023-48322-y)
Supplement: Supplementary file 1 — Supplementary Information. [file 41598_2023_48322_MOESM1_ESM.pdf]

Supplement data

Fabrication of conductive Ag/AgCl/Ag nanorods ink on flexible Laser-induced graphene electrodes for non-enzymatic glucose detection

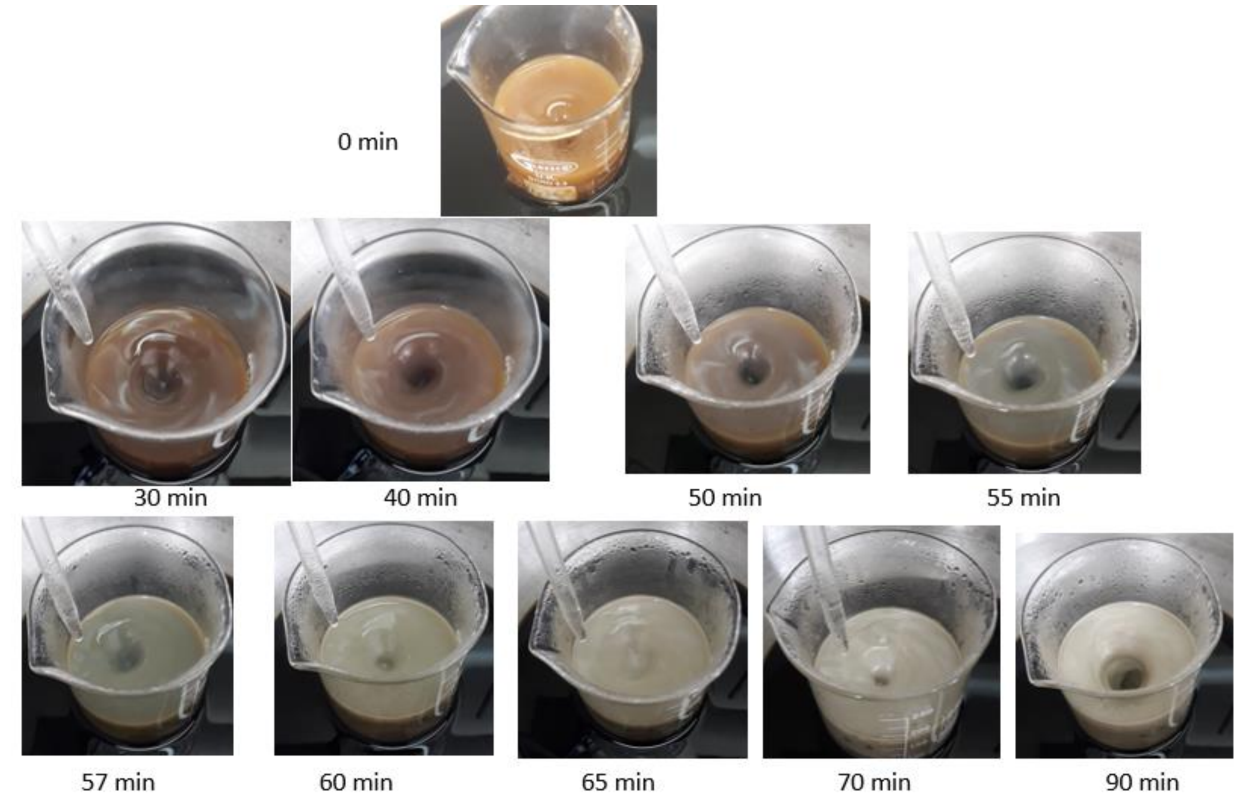

Fig. S1. The change in color of Ag/AgCl/Ag nanorods quasi reference at various times.

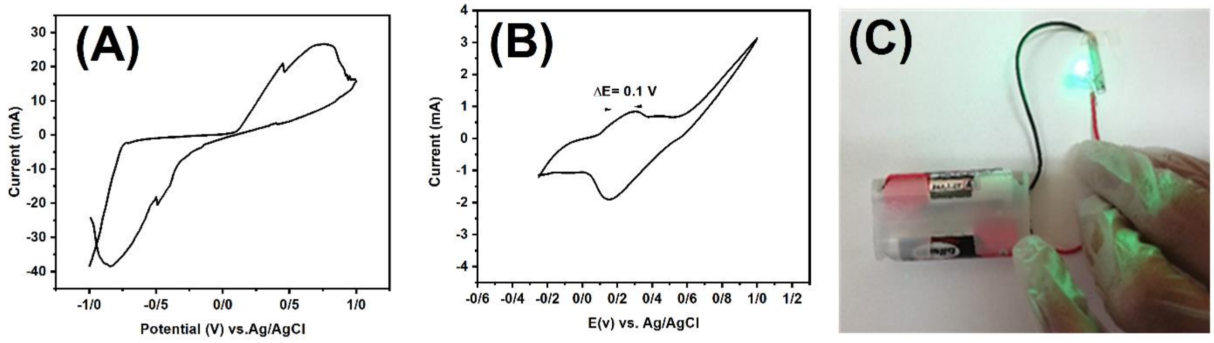

Fig. S2. Cyclic voltammetry of Ag/AgCl/Ag ink in (A) $K_3[Fe(CN)_6]$, and $K_4[Fe(CN)_6]$ (5 mM), KCl (0.1 M) (B) $K_3[Fe(CN)_6]$ (5 mM) and 0.1M KNO_3 in PBS (pH 7.4) solution at 50 $mV.s^{-1}$, (C) Optical images of LED-connected Ag/AgCl/Ag electrode lines on PET.

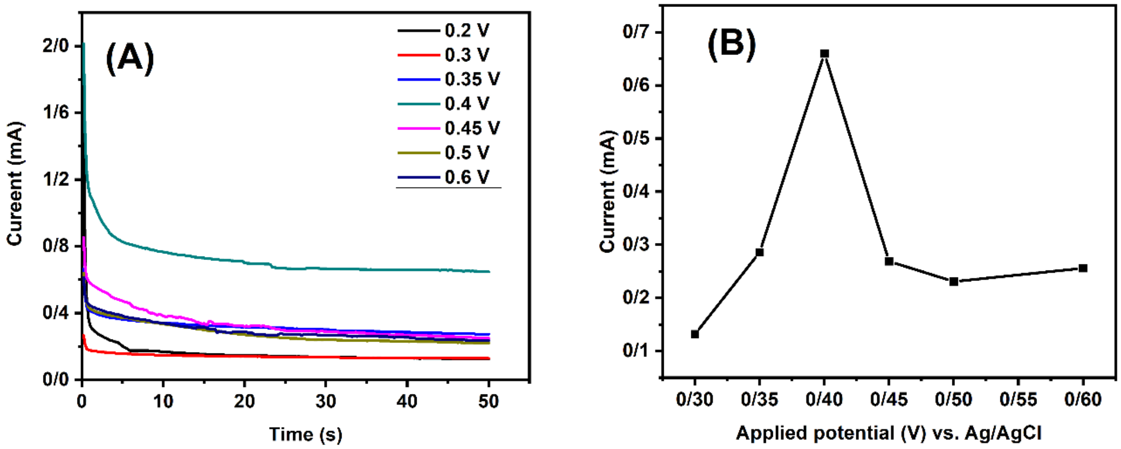

Fig. S3. (A) The amperometric curve of different applied potentials and (B) calibration curve of applied potential vs current response of Ag/AgCl/Ag on LIG electrode.

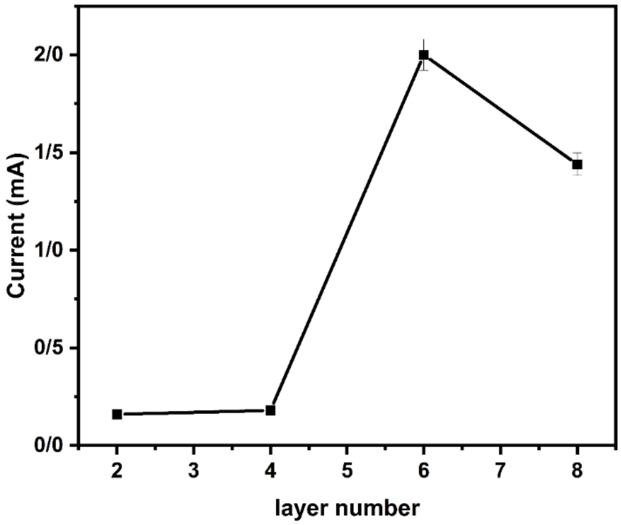

Fig. S4. The calibration curve of different layer numbers vs the current response.

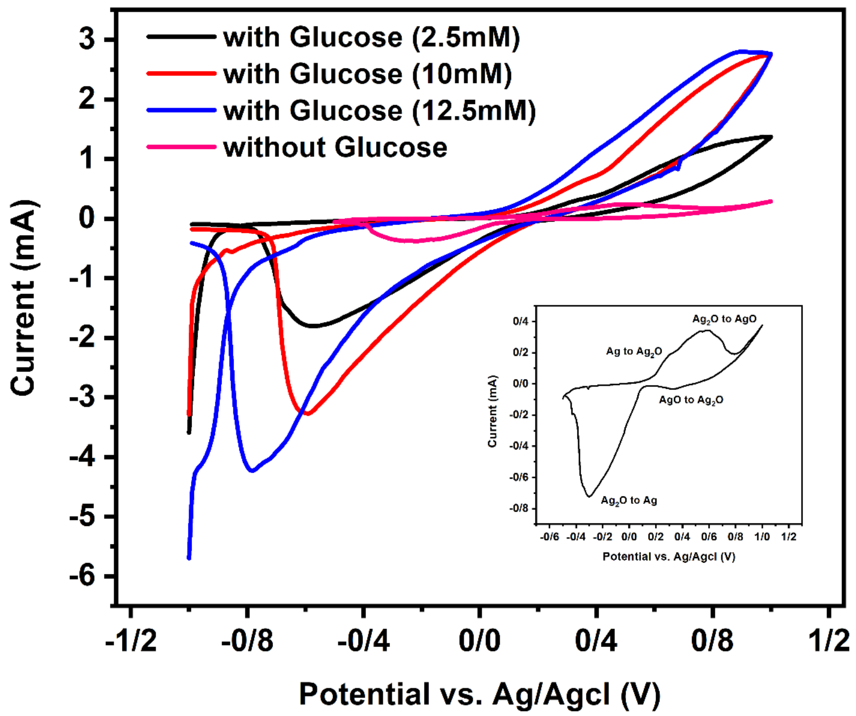

Fig .S5. The cyclic voltammetry of glucose sensor was recorded in 0.1 M NaOH with a scan rate of 25 mV.s^{-1}, and (insert) the cyclic voltammetry in the absent glucose.

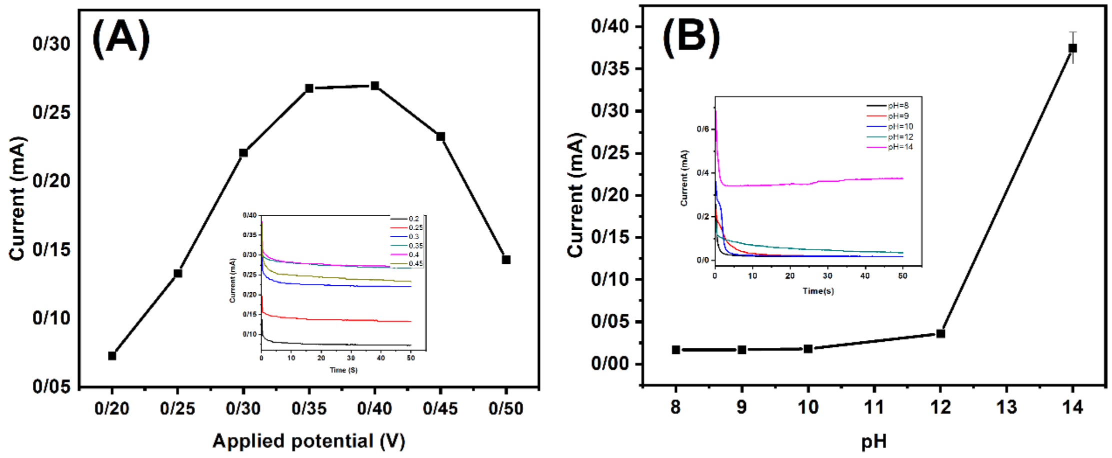

Fig. S6. The calibration curve of (A) applied potential (B) pH vs current response of electrode and (insert) Amperometric responses for different applied potential and pH represent at a potential at +400 mV vs. Ag/AgCl in 0.1 mM glucose.

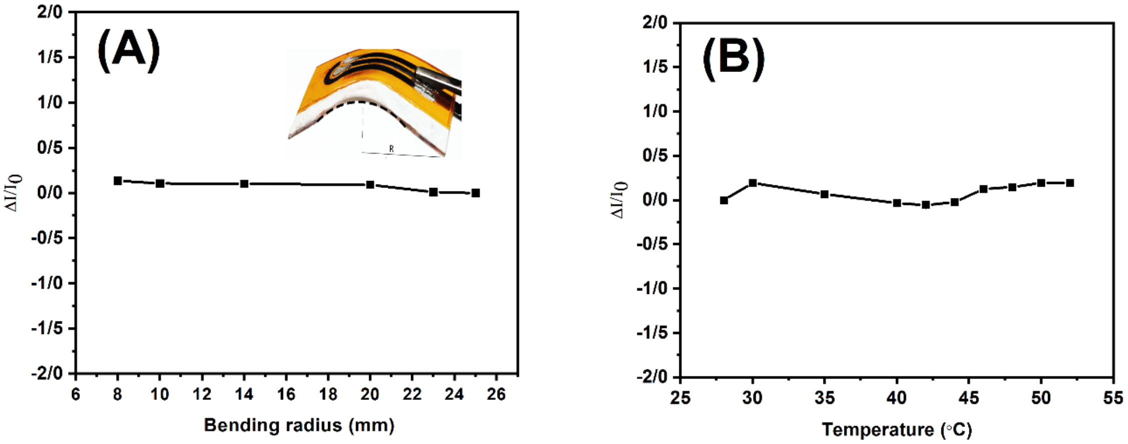

Fig. S7. The effect of (A) bending (at room temperature) and (B) temperature on the response of the biosensor at +400 mV vs. Ag/AgCl in 0.10 M NaOH solution with 0.1 M glucose.

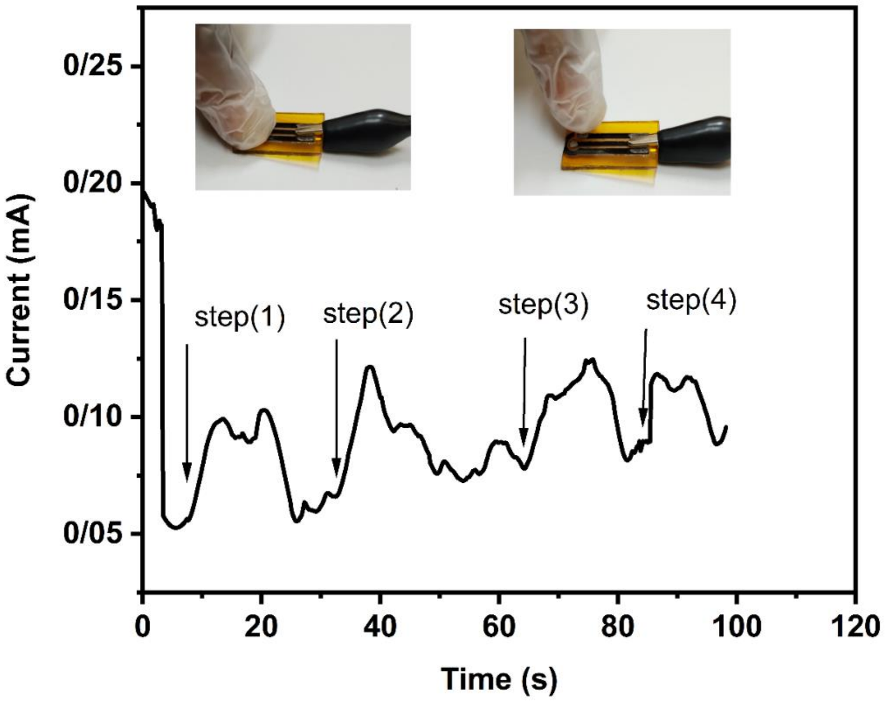

Fig S8. The Applications of strain sensors. Real-time monitoring of human motion by detecting current change during finger touching, (inset) the digital photos of a glucose sensor attached to an electrode for testing with 5 mM glucose in 0.1M NaOH.

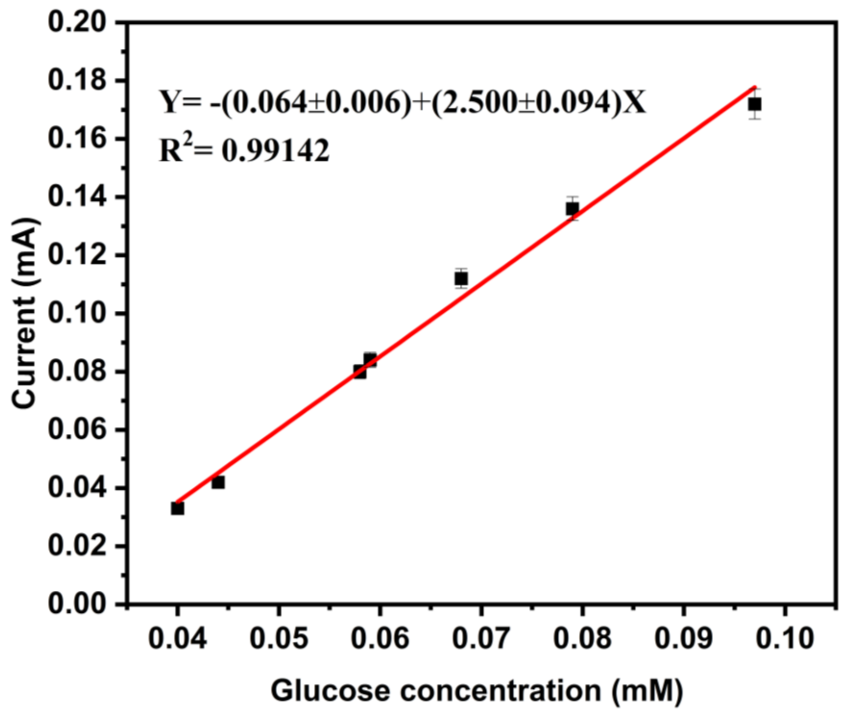

Fig S9. Real sample analysis of glucose in human serum samples.

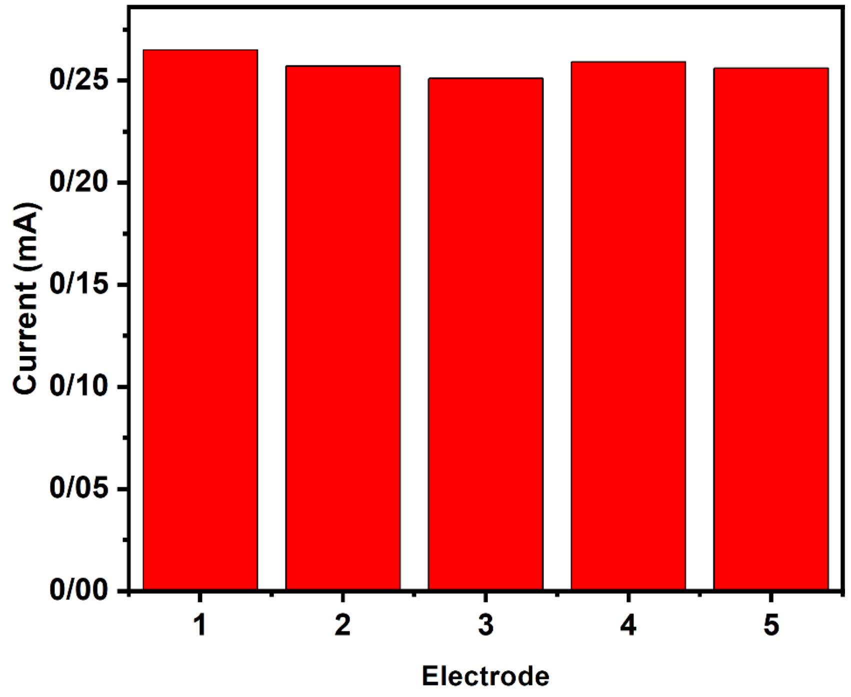

Fig. S10. The response of five Ag/AgCl/Ag NRs/LIG electrodes to 0.1 M glucose in 0.1 M NaOH solution.

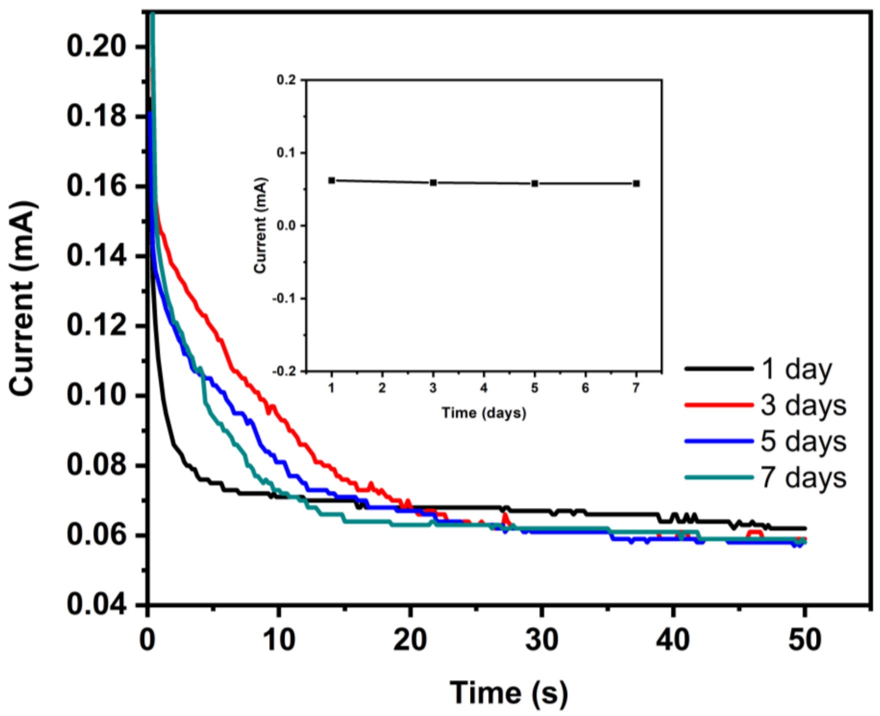

Fig S11. The stability of the biosensor with 5mM glucose for one week, with the corresponding current-time plot of the amperometric response shown in the inset.
